# Propagating pluripotency – The conundrum of self‐renewal

**DOI:** 10.1002/bies.202400108

**Published:** 2024-08-23

**Authors:** Austin Smith

**Affiliations:** ^1^ Living Systems Institute University of Exeter Exeter UK

**Keywords:** embryogenesis, embryonic stem cells, epiblast, pluripotency, self‐renewal, signaling, stem cells

## Abstract

The discovery of mouse embryonic stem cells in 1981 transformed research in mammalian developmental biology and functional genomics. The subsequent generation of human pluripotent stem cells (PSCs) and the development of molecular reprogramming have opened unheralded avenues for drug discovery and cell replacement therapy. Here, I review the history of PSCs from the perspective that long‐term self‐renewal is a product of the in vitro signaling environment, rather than an intrinsic feature of embryos. I discuss the relationship between pluripotent states captured in vitro to stages of epiblast in the embryo and suggest key considerations for evaluation of PSCs. A remaining fundamental challenge is to determine whether naïve pluripotency can be propagated from the broad range of mammals by exploiting common principles in gene regulatory architecture.

## Graphical abstract

Self‐renewal of pluripotent stem cells (PSCs) is achieved in signaling environments that stall developmental progression while permitting proliferation. Naïve PSCs corresponding to early epiblast are captured in a signal‐restricted milieu. In contrast, specific growth factor stimulation is necessary to sustain formative or primed PSCs that represent transitional and mature epiblast.

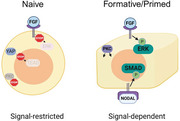

## ORIGIN OF PLURIPOTENT STEM CELLS

Pluripotency is the flexibility to be guided by extrinsic signals to form any of the major developmental lineages of the organism. In mammals, pluripotency ends at gastrulation; subsequent progenitor and stem cells are lineage restricted. After the early embryo, production of distinct tissue lineages from a common origin occurs only in a type of germline tumor, teratocarcinoma.^[^ ^1^ ^]^ Teratocarcinomas also contain proliferative undifferentiated cells, termed embryonal carcinoma (EC). The establishment of an inbred mouse strain that developed spontaneous teratocarcinoma^[^ ^2^ ^]^ allowed systematic investigation of EC cells. EC cells were found to be capable at the single cell level of reforming teratocarcinomas in vivo.^[^ ^3^ ^]^ They could be expanded from explant cultures and established as cell lines.^[^ ^1^ ^]^ In vitro EC cells were demonstrated to undergo multilineage differentiation after clonal propagation.^[^ ^4^, ^5^ ^]^ They are thus the first described pluripotent stem cells (PSCs). Notably, aggregates of EC cells in suspension culture differentiated into structures termed embryoid bodies that showed features of early embryos, predating contemporary gastruloid and other embryoid models.^[^ ^5^ ^]^

To explore the relationship between pluripotency in the embryo and in teratocarcinomas, early mouse embryos were grafted to adult tissues.^[^ ^6^, ^7^ ^]^ A proportion of grafts gave rise to teratocarcinomas. EC cells could be derived from these tumors, supporting the idea of a connection to embryo pluripotency. However, EC cell lines are aneuploid,^[^ ^1^ ^]^ likely as a consequence of selection for growth in tumors. They also vary in their growth and differentiation properties.^[^ ^1^ ^]^ The goal of a non‐transformed PSC motivated attempts to propagate cells directly from the embryo. In 1981, these efforts came to fruition when stem cells were captured in culture without an intermediate stage of tumor growth.^[^ ^8^, ^9^ ^]^ Initially it was debated whether these pluripotent embryonic stem (ES) cells had transformed into EC in vitro or remained developmentally and genetically intact. The dispute was emphatically resolved by reintroduction of ES cells to the early embryo and demonstration of contributions to all tissues in chimeric mice, including production of functional gametes and derivative healthy offspring.^[^ ^10^ ^]^ The power of genetic manipulation of ES cells for transmission into the mouse germline was rapidly harnessed to engineer sophisticated genome modifications in mice, transforming reverse genetics and functional genomics research.^[^ ^11^, ^12^ ^]^

Interestingly, although they are genetically normal, ES cells form teratocarcinomas if grafted to adult mice. It has not been determined; however, whether the EC cell component of those tumors remains diploid. Crucially, ES cells, unlike EC cells, do not give rise to tumors in embryo chimeras. The embryonic signaling environment constrains an intrinsic tumorigenic potential in PSCs by enforcing differentiation.

## THE SELF‐RENEWAL PARADOX

ES cells have formidable self‐renewal capacity. They have been shown to retain full developmental potential after serial sub‐cloning entailing continuous cell replication every 12 h for several months.^[^ ^13^ ^]^ Yet in the mouse embryo the entire period of pluripotency extends no longer than 5 days. Furthermore, pluripotent epiblast in the embryo undergoes dramatic molecular and morphological changes during this period. Neither self‐renewal nor hierarchical organization, the hallmark features of adult stem cell systems, is apparent.

The contradiction between transience in the embryo and unlimited self‐renewal in vitro questioned the fundamental nature and relevance of ES cells. Are they a serendipitous byproduct of a particular inbred genetic background, or a consequence of genetic or epigenomic mutability in cell culture? Or do they emanate directly from the gene regulatory network underpinning pluripotency? Resolving this issue required elucidating the molecular basis of ES cell derivation and propagation, characterizing their relatedness to resident epiblast cells, and determining the generality of PSC propagation across mammals.

## THE MOUSE EMBRYONIC STEM CELL PARADIGM

The original derivation of ES cells was achieved by culturing inner cell masses (ICMs) from mouse blastocysts in conditions that had been optimized empirically for maintaining pluripotency in EC cells.^[^ ^14^ ^]^ These conditions comprise co‐culture with a “feeder layer” of mitotically inactivated embryo fibroblasts and use of a fetal calf serum batch selected for supporting colony formation by EC cells. Culture on feeders with screened serum enables robust propagation of diploid ES cells from the inbred mouse strain *129*. However, these conditions have limited efficacy for other mouse strains. Moreover, reliance on feeders and serum introduces multiple variables that hinder mechanistic studies. Fortunately, it was appreciated relatively quickly that the major contribution of feeders was to provide a specific cytokine, leukemia inhibitory factor (LIF).^[^ ^15^, ^16^, ^17^ ^]^ Supplementation with recombinant LIF can replace feeders in derivation and propagation of germline competent ES cells.^[^ ^18^ ^]^

Without serum, however, ES cells differentiate even in the presence of LIF. This differentiation is predominantly into neuroectoderm^[^ ^19^ ^]^ and can be suppressed by bone morphogenetic protein (BMP). LIF and BMP together enable feeder and serum‐free propagation of ES cells, providing the first defined medium for maintaining pluripotency.^[^ ^20^ ^]^ This combination, however, does not overcome the barrier to ES cell derivation from diverse mouse strains or from other rodents.

In all of the above culture conditions, the great majority of cells are compact and undifferentiated, although some differentiated morphologies appear at the periphery of colonies. The ES cell state was therefore considered to be essentially homogeneous with occasional cells escaping into differentiation. However, closer inspection by immunostaining and live cell reporter studies revealed that expression of multiple transcription factors is heterogeneous and dynamic.^[^ ^21^, ^22^, ^23^ ^]^ Heterogeneity for some factors is associated with differing clonogenic potency.^[^ ^24^, ^25^ ^]^ Dynamic fluctuations in transcription factor expression occur during the unidirectional trajectory of pluripotency in the embryo have not been described. Metastability observed in ES cells may result from a stop‐go environment of conflicting self‐renewal and differentiation signals in vitro.^[^ ^26^ ^]^

What is the differentiation signal(s)? Key insight came from the findings that the autocrine growth factor FGF4 promotes ES cell differentiation and that this is mediated primarily by the ERK1 and ERK2 signaling cascade.^[^ ^27^, ^28^ ^]^ This led to the realization that differentiation can largely be suppressed by preventing activation of ERK signaling with small molecule inhibitors of the upstream kinases MEK1 and MEK2. MEK inhibition alone is not sufficient to sustain ES cell self‐renewal without LIF, however. To replace LIF, a second small molecule, the glycogen synthase kinase‐3 (GSK3) inhibitor CHIR99021 (CH), is required. GSK3 inhibition both simulates activation of the canonical Wnt pathway and promotes cell growth.^[^ ^29^ ^]^ The two inhibitor combination, 2i, is sufficient to support ES cell colony formation from single cells without feeders or serum^[^ ^30^ ^]^ (Table 1). ES cells derived and continuously propagated in 2i are competent to form germline chimeras. The robustness of self‐renewal, as measured in colony forming and proliferation assays, is further enhanced by addition of LIF.^[^ ^31^, ^32^, ^33^ ^]^ The combination of 2iLIF has been shown to support ES cell generation from multiple strains and subspecies of mice,^[^ ^30^, ^34^ ^]^ and moreover enables derivation from rat embryos.^[^ ^35^, ^36^ ^]^

**TABLE 1:** Signaling pathway modulators used for pluripotent stem cell (PSC) self‐renewal.^[^ ^30^, ^68^, ^70^, ^111^, ^115^, ^118^ ^]^

		Naïve		Formative/primed	
		Mouse	Human	Mouse	Human
**Pathway inhibitor**	MEKi/ERKi	+	+	−	−
	PKCi^a^	±	+	−	−
	TNKSi^b^	−	+	−	−
	WNTi^c^	−	−	±	±
	SRCi	±	±	−	−
	FGFRi	±	±	−	−
**Pathway activator**	GSK3i	+	−	−	−
**Growth factor**	LIF	+	±	−	−
	FGF	−	−	+	+
	Activin/TGFβ	−	±	+	+
**Other**	Serum/KSR	−	−	±	±
	Geltrex^d^	−	+	−	+
	Feeders	−	+	−	±
	5% O_2_	±	+	−	−

+, strongly promotes self‐renewal; ±, alternative or optional; −, not required or detrimental.

^a^ Gö6983 inhibits both canonical and atypical isoforms of protein kinase C.

^b^ Tankyrase inhibitors such as XAV939 and IWR1 inhibit both the canonical WNT pathway and the YAP/TEAD pathway.

^c^ WNT inhibitors include tankyrase inhibitors and PORCN inhibitors such as IWP‐2 and Wnt‐C59.

^d^ ECM preparation, also known as Matrigel.

These findings established that ES cell derivation is not a quirk of a particular inbred strain of mouse but is a more general feature of rodent embryos. Furthermore, in 2iLIF the transition between pre‐implantation epiblast and ES cell appears seamless; single epiblast cells will begin to self‐renew and give rise to ES cell lines at high efficiency, appearing to rule out requirement for genetic alteration or major epigenomic reprogramming.^[^ ^37^ ^]^ These ES cells retain the global gene expression profile and the functional capacity of early epiblast cells. Furthermore, expression of key transcription factors is relatively homogeneous. Thus, ES cells in 2iLIF have been proposed to reside in a ground state, denoting an equivalence group of shared identity and potency with no bias for somatic fates.^[^ ^38^, ^39^, ^40^ ^]^ Furthermore, since the discovery of induced pluripotency by molecular reprogramming,^[^ ^41^ ^]^ it has been shown that 2iLIF components individually and collectively facilitate generation of fully reprogrammed mouse iPSCs.^[^ ^42^, ^43^, ^44^, ^45^ ^]^ It should be noted, however, that 2iLIF is not without limitation because genomic imprints are susceptible to erasure, especially if cultures are allowed to overgrow.^[^ ^46^, ^47^, ^48^ ^]^

## SIGNALING LOGIC FOR ES CELL SELF‐RENEWAL

**FIGURE 1:**
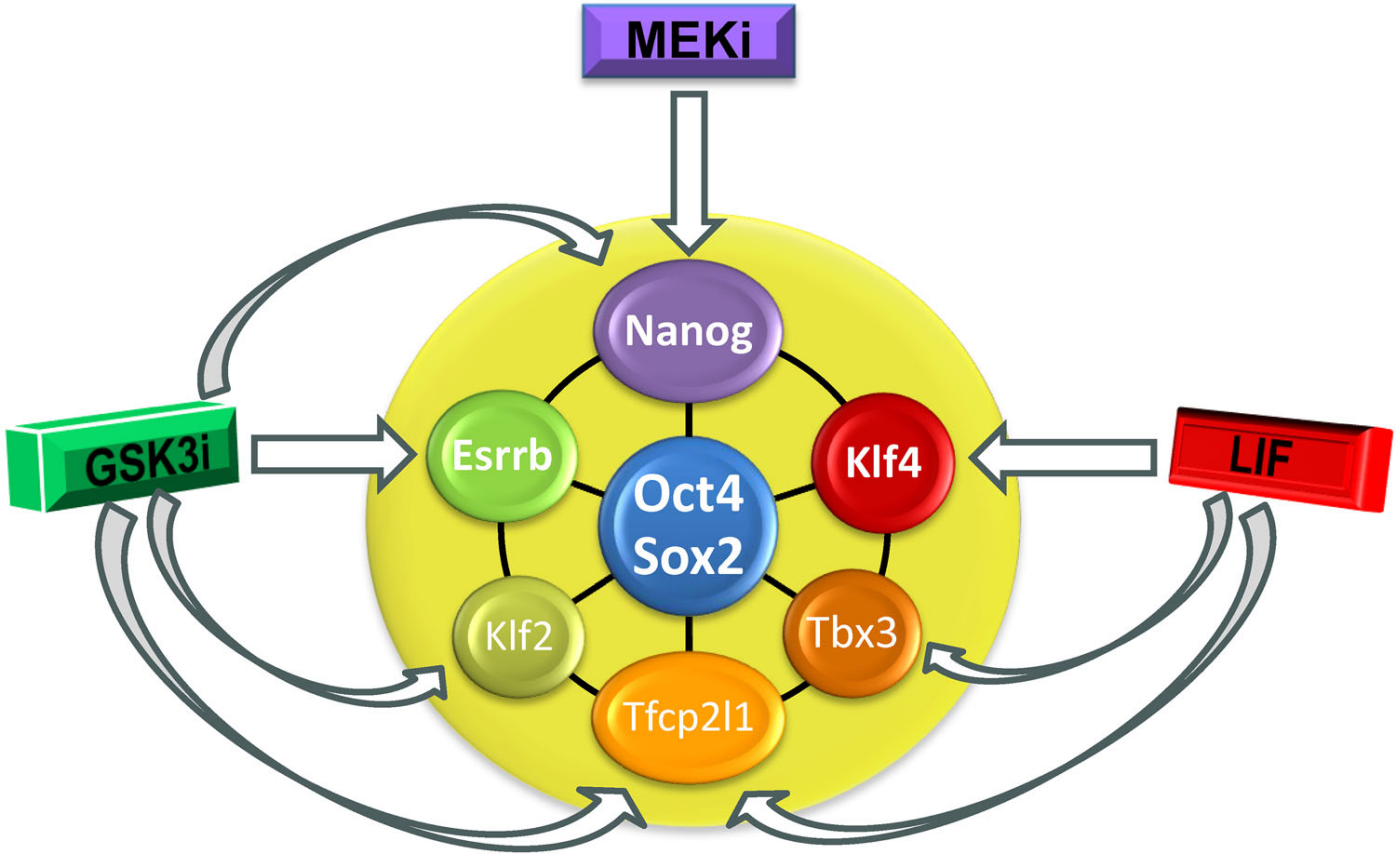
Signaling and transcription factor network for mouse ES cell self‐renewal. The three components of 2iLIF^[^ ^30^, ^33^ ^]^ (MEK/ERK inhibition by PD0325901, inhibition of GSK3 by CHIR99021,^[^ ^29^ ^]^ and the cytokine leukemia inhibitory factor) sustain a cooperative transcription factor network that determines naïve ES cell identity. This combination of transcription factors is found only in naïve epiblast, ES cells, primordial germ cells, and EC cells. The outer ring of factors is regulated directly by 2iLIF. These factors are extinguished during formative transition (although Nanog is re‐expressed in EpiSCs), while generic pluripotency factors Oct4 and Sox2 are maintained. Arrows indicate experimentally validated major targets of individual components of 2iLIF.^[^ ^50^ ^]^ (For a related computational network model see ref. [31]). LIF and GSK3 inhibition promote transcription of indicated transcription factors, whereas MEK inhibition maintains Nanog protein level.^[^ ^132^ ^]^ EC, embryonal carcinoma; ES, embryonic stem; LIF, leukemia inhibitory factor.

The ES cell gene regulatory network is governed by a specific set of transcription factors that act in overlapping and cooperative fashion to sustain ES cell identity and potency.^[^ ^49^, ^50^ ^]^ This suite of transcription factors, principally comprising Nanog, Esrrb, Klf4, Klf2, Tbx3, Tfcp2l1, and Gbx2^[^ ^31^ ^]^ are co‐expressed only in ES cells and the epiblast of the pre‐implantation embryo. They act in conjunction with the more broadly expressed core pluripotency factors Oct4 and Sox2. The components of 2iLIF act to promote and maintain expression of the ES cell‐specific transcription factors^[^ ^31^, ^49^ ^]^ (Figure 1).

Is there a developmental logic underlying the ability of 2iLIF to sustain pluripotency? The differentiation suppressing effect of MEK/ERK inhibition is consistent with genetic perturbations of the FGF pathway in the embryo^[^ ^51^, ^52^, ^53^ ^]^ and with selective expansion of the epiblast in preimplantation embryos treated with MEK inhibitor.^[^ ^54^, ^55^ ^]^ On the other hand, absence of zygotic LIF or its essential signal transducer Stat3^[^ ^56^ ^]^ have little effect on the embryo until post‐implantation stages^[^ ^49^, ^57^ ^]^ (although maternal/zygotic deletion of Stat3 results in early ICM collapse^[^ ^58^ ^]^). Likewise, deletion of porcupine to abolish Wnt signaling does not disrupt establishment of blastocyst lineages including epiblast.^[^ ^59^ ^]^ Therefore, neither of these pathways is uniquely required in the early epiblast during normal development. However, there is a particular context in which both become essential. This is during diapause, a facultative adaptation whereby implantation is delayed and embryos become dormant at the late blastocyst stage.^[^ ^60^ ^]^ Epiblast cells are largely quiescent during diapause, but their identity and potency are fully maintained. Ablation of either LIF/Stat3 or WNT pathways causes collapse of the epiblast compartment in diapause embryos.^[^ ^61^, ^62^ ^]^ Thus, these signals function to prolong the normally transient state of early epiblast. It is of note that the original derivation of ES cells was from diapause blastocysts^[^ ^8^ ^]^ and diapause has generally been considered an advantageous starting condition for ES cell derivation.^[^ ^63^ ^]^

Self‐renewal of ES cells is in essence the pausing of developmental progression while sustaining proliferation. Diapause induced growth arrest is relieved in culture, likely by nutrients that reactivate mTOR signaling and Myc activity.^[^ ^64^, ^65^ ^]^ The natural facility for diapause in mice may therefore account for the relative ease with which ES cells can be established from pre‐implantation epiblast in this species and provides an explanation for the signaling dependencies. Conversely, the absence of diapause in most mammals may hinder derivation of PSCs equivalent to mouse ES cells or mean that alternative signaling environments are required.

## ALTERNATIVE PLURIPOTENT STEM CELLS FROM HUMAN AND OTHER MAMMALS

In 1998, Thomson et al. achieved the landmark of establishing PSCs from human embryos.^[^ ^66^ ^]^ As for the original mouse ES cells, derivation was enabled by feeders and serum. It was initially assumed that mouse and human PSCs should be counterparts. However, with increasing characterization multiple differences became apparent.^[^ ^67^ ^]^ Notably, human PSCs are epithelial‐like and cannot be propagated in any of the defined conditions for mouse ES cells. Instead, their self‐renewal is supported by FGF together with activin or TGFβ^[^ ^68^, ^69^, ^70^ ^]^ (Table 1). Species‐specific attributes of pluripotency were invoked as an explanation for these and other differences. An alternative proposal was that mouse and human PSCs represented epiblast at different developmental stages. The latter idea was substantiated when human PSC culture conditions were applied to mouse post‐implantation egg cylinder epiblast. ES cells cannot be derived from this stage, but stem cell lines were obtained using FGF and activin.^[^ ^71^, ^72^ ^]^ These post‐implantation epiblast‐like stem cells, EpiSCs, are phenotypically distinct from ES cells. Although pluripotent and able to form teratocarcinoma, EpiSCs do not colonize chimeras when introduced to pre‐implantation embryos. Consistent with sequential developmental staging, ES cells can be differentiated into EpiSCs but EpiSCs do not readily revert to ES cells.^[^ ^73^ ^]^

Thus, distinct PSC types can be derived that correspond to early and late stages of pluripotency in the mouse embryo. In the case of human PSCs, transcriptome analyses have now confirmed similarity to post‐implantation epiblast rather than blastocyst stage epiblast.^[^ ^74^, ^75^ ^]^ This can be explained by continued development of ICM explant cultures to later stage epiblast prior to PSC establishment.^[^ ^76^ ^]^ To reflect their more advanced developmental stage poised for gastrulation, EpiSCs and conventional human PSCs are given the general descriptor “primed”.^[^ ^77^ ^]^ This contrasts with the earlier, specification‐free, status of mouse ES cells, which is designated “naïve”.

In FGF and activin (or TGFβ), mouse and human PSC cultures display variable expression of early lineage markers. Heterogeneity and primitive streak‐like differentiation in primed PSCs are reduced by inhibition of WNT using small molecules such as XAV939, IWR‐1, or IWP2.^[^ ^78^, ^79^ ^]^ Even in this culture condition, it is problematic to assign a specific embryonic counterpart to primed PSC cultures,^[^ ^79^, ^80^ ^]^ not least because embryo epiblast undergoes regional specification during expansion and cannot be considered as a unitary cell type.^[^ ^80^ ^]^ Moreover, the primed PSC phenotype appears to be maintained by a balance of conflicting signals. Activin and TGFβ serve as surrogates for Nodal, which plays a central role in primitive streak induction and mesoendoderm specification in the embryo,^[^ ^81^ ^]^ while FGFs are variously implicated in proliferation, migration, and differentiation.^[^ ^82^ ^]^ Conflicting differentiation and proliferation signals may translate into metastability.^[^ ^83^ ^]^

Despite the challenges of heterogeneity and variability which hinder standardization, remarkable progress has been made in advancing human primed PSCs and iPSCs for translational applications in disease modeling and cell therapy.^[^ ^84^ ^]^ For these purposes, naïve PSCs are not required and would only offer an advantage if shown to be less variable.

Interestingly, the combination of activin (or TGFβ), FGF, and Wnt inhibitor, without serum factors or feeders, can support propagation of primed‐type PSCs from rodents, rabbits, livestock mammals, and primates.^[^ ^85^, ^86^, ^87^ ^]^ The ability to establish PSCs related to egg cylinder or embryonic disk epiblast from a range of mammals in a standard signaling milieu contrasts with the persisting elusiveness of naïve PSCs. The common feature of expansion prior to gastrulation may predispose for continuous propagation and stem cell derivation from epithelialized epiblast.

## CAPTURE OF HUMAN NAÏVE PLURIPOTENCY

For many years naïve PSCs were unique to mice and rats.^[^ ^88^ ^]^ However, realization that mouse EpiSCs could efficiently be converted to ES cells by transient expression of naïve pluripotency factors and transfer to 2iLIF^[^ ^73^ ^]^ stimulated approaches to reset human primed PSCs. Naïve‐type colonies could be obtained, but they were not expandable in 2iLIF. By screening candidate signaling inhibitors continuous propagation of reset human PSCs was eventually achieved.^[^ ^89^, ^90^ ^]^ These cells exhibit a range of anticipated naïve attributes. DNA hypomethylation and activation of both X chromosomes in female cells are hallmarks of pre‐implantation naïve epiblast manifest in naïve PSCs.^[^ ^91^ ^]^ Since chimera contribution cannot ethically be tested in humans, transcriptome identity is a primary criterion. Global transcriptome relatedness to human pre‐implantation epiblast with distinction from both post‐implantation epiblast and primed PSCs has been verified in multiple studies.^[^ ^74^, ^75^, ^91^, ^92^ ^]^ Furthermore, naïve PSCs can be established directly from dissociated human ICMs without the intervening explant maturation required to derive primed PSCs.^[^ ^93^ ^]^

A striking feature of human naïve PSCs is that they do not differentiate directly into somatic lineages. On withdrawal from self‐renewal conditions, the competence to generate somatic lineages develops over several days.^[^ ^94^ ^]^ This formative process is called capacitation (see below). After capacitation, resulting primed‐type cells can be stably propagated in activin and FGF‐based medium like conventional PSCs and readily be induced to differentiate. An area for further investigation is whether newly capacitated PSCs may exhibit differences in differentiation responsiveness or consistency compared to conventional primed PSCs.

Naïve induced PSCs can readily be generated by molecular reprogramming.^[^ ^41^ ^]^ They are recovered at similar efficiencies to primed iPSCs using the same reprogramming factors, with the outcome dictated by the signaling milieu^[^ ^95^, ^96^ ^]^ (Table 1). Systematic comparison using multiple pairs of isogenic naïve and primed iPSCs will be necessary to determine whether the largely erased epigenome status of naïve cells may yield any consistent improvement in multilineage differentiation efficiency.

Human naïve PSCs exhibit a differentiation property that was not anticipated. Unlike mouse ES cells they are able to form blastocyst stage trophoblast.^[^ ^97^, ^98^ ^]^. Mouse ES cells obey the lineage restriction observed for mouse epiblast in vivo^[^ ^99^, ^100^ ^]^ and do not normally generate trophoblast, either in chimeras or by in vitro differentiation.^[^ ^101^, ^102^ ^]^ In contrast, human naïve PSCs can readily be differentiated into trophoblast by altering the signaling environment. Naïve epiblast cells taken directly from the human blastocyst can also be stimulated to form trophoblast.^[^ ^98^ ^]^ Thus, plasticity to form trophoblast reflects a species‐difference in developmental timing of lineage restriction between mouse and human. Whether naïve epiblast in other mammals retains trophoblast potential and why this feature is absent in rodents are areas for future investigation.

The ability of human naïve PSCs to form trophoblast, and also the second extraembryonic lineage, hypoblast, can be harnessed to form blastocyst‐like structures termed blastoids.^[^ ^103^, ^104^ ^]^ Blastoids are similar in shape, size, and cellular composition to blastocysts. They form over 3–4 days from naïve PSCs clustered in microwells. Differentiating trophoblast cells segregate and form an epithelial layer outer layer. Fluid uptake across the trophoblast epithelium leads to cavitation. At the same time, the internal cells resolve into epiblast and hypoblast compartments. Extended cultures of blastoids can exhibit some features of peri‐implantation development, albeit at low efficiency.^[^ ^103^, ^104^, ^105^ ^]^ Thus, the plasticity of human naïve PSCs and the capacity to form blastoids hold the promise of readily accessible, experimentally tractable, and scalable platforms for studying early human embryogenesis, infertility, and developmental abnormalities.^[^ ^106^, ^107^ ^]^

## SIGNALING LOGIC FOR HUMAN NAÏVE PSC SELF‐RENEWAL

The original formulations for human naïve PSCs, 5iLA^[^ ^108^ ^]^ and t2iLGö,^[^ ^90^ ^]^ rely on inhibition of FGF/ERK signaling and include both LIF and a GSK3 inhibitor. Rho‐associated kinase inhibitor is added to improve viability of dissociated cells during passaging.^[^ ^109^ ^]^ In 5iLA, which contains RAF and SRC inhibitors, cells acquire chromosomal abnormalities at early passages indicating untoward selective pressure.^[^ ^91^ ^]^ Cultures show better karyotype stability in t2iLGö and a more recent version, PXGL.^[^ ^110^, ^111^ ^]^ PXGL comprises the MEK inhibitor PD0325901 together with XAV939, an inhibitor of tankyrase,^[^ ^112^ ^]^ Gö6983 a pan‐protein kinase C (PKC) inhibitor also used in t2iLGö, and LIF^[^ ^113^ ^]^ (Table 1). The key difference from t2iLGö is introduction of XAV939. Naïve PSCs in t2iLGö or PXGL appear similar but in the latter can be grown to larger colony size before passaging and show a more homogeneous single cell transcriptome profile. Dispensability of GSK3 inhibition for human naïve PSCs^[^ ^29^, ^114^ ^]^ is in line with the finding that key downstream effectors in mouse ES cells, Tcf7l1 and Esrrb,^[^ ^29^, ^114^ ^]^ are barely expressed in human naïve epiblast or PSCs.^[^ ^90^ ^]^ LIF is typically included in human naïve PSC medium, although its effects appear marginal. The contrasting ability of Wnt and LIF signaling to sustain mouse ES cells, but not human naïve PSCs may stem from their rodent‐specific roles in diapause discussed above.

**FIGURE 2:**
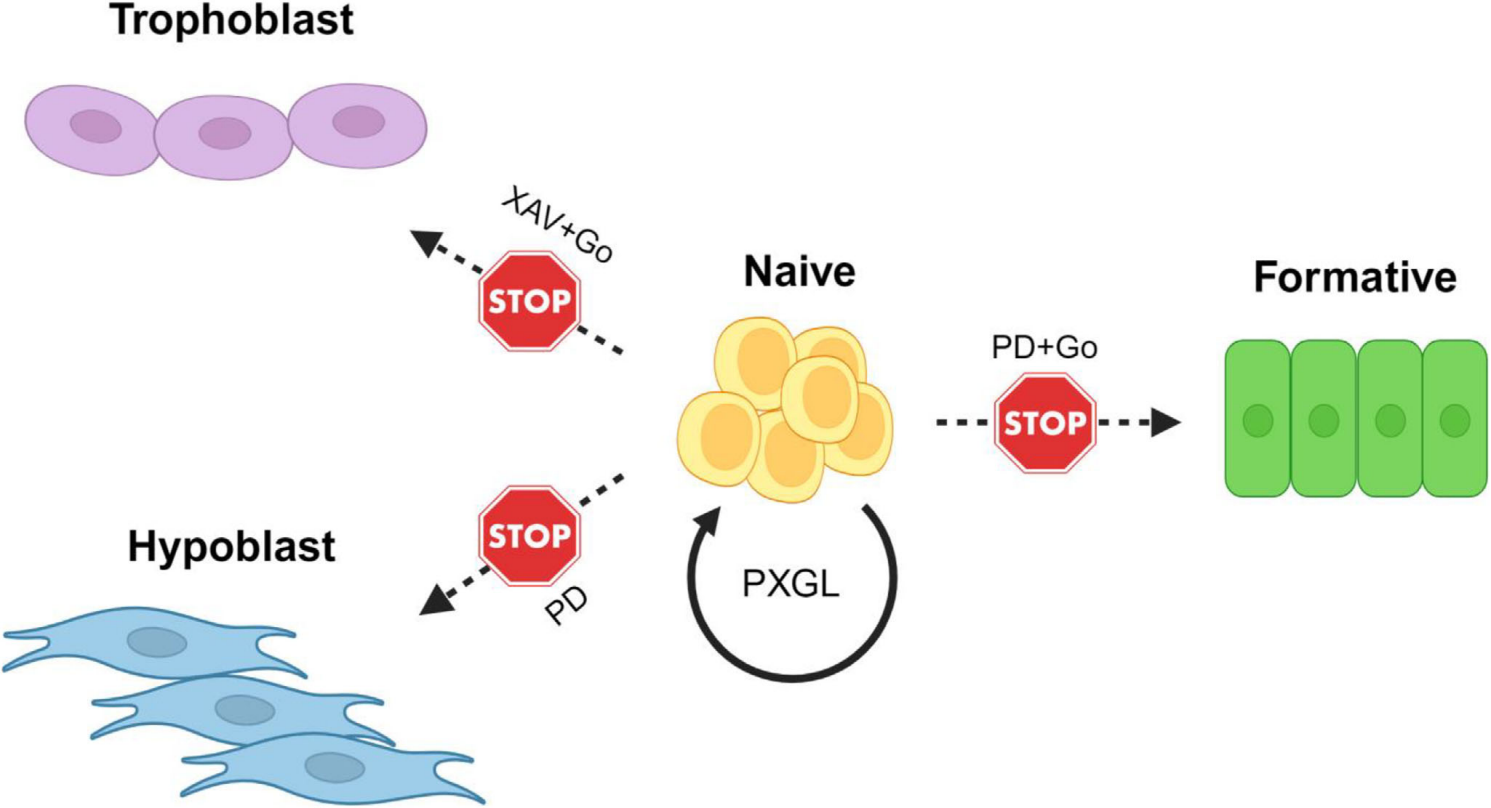
Signaling context for human naïve PSC self‐renewal. Human naïve PSCs are maintained in PXGL medium. The propensity for trophectoderm and hypoblast differentiation demands additional inhibitor requirements compared with mouse ES cells. MEK inhibitor PD0325901 (PD) together with PKC inhibitor Gö6983 (Go) block formative transition. PD also prevents hypoblast differentiation, while tankyrase inhibitor XAV939 (XAV) together with Go suppresses trophoblast differentiation. The importance of LIF for human naïve PSCs is uncertain. Created with BioRender.com. ES, embryonic stem; LIF, leukemia inhibitory factor; PKC, protein kinase C; PSC, pluripotent stem cell.

FGF/ERK inhibition is a core requirement for human naïve PSC self‐renewal,^[^ ^115^ ^]^ as for mouse ES cells. It impedes both formative transition and hypoblast specification (Figure 2). In human naïve PSCs, however, FGF/ERK blockade also enables trophectoderm differentiation.^[^ ^98^ ^]^ Tankyrase inhibition counteracts trophectoderm induction. This is not dependent on blockade of canonical WNT signaling, the best known effect of XAV939, because β‐catenin deficient naïve PSCs remain dependent.^[^ ^116^ ^]^ Instead the key effect of tankyrase inhibition is to suppress activation of YAP and its nuclear translocation.^[^ ^117^ ^]^ Resulting low levels of nuclear YAP are insufficient for the interaction with TEAD transcription factors required to initiate the trophectoderm programme.^[^ ^116^ ^]^ Gö6983 was identified as a facilitator of mouse ES cell self‐renewal, an effect attributed to inhibition of atypical PKC.^[^ ^118^, ^119^ ^]^ A rationale is that aPKC inhibition prevents establishment of apical polarity, necessary for epithelialization as cells exit naïve pluripotency.^[^ ^120^ ^]^ In human naïve PSCs such action may contribute to suppression of epithelial trophectoderm differentiation as well as formative transition. However, inhibition of other PKC isoforms by Gö6983 may also play a role, either by reducing YAP activity^[^ ^121^, ^122^ ^]^ or other effects.

Feeder cells are currently used for reliable propagation of human naïve PSCs. Without feeders, PSCs remain in a naïve state in PXGL, but their growth slows after 3–4 passages. With or without feeders, naïve PSC expansion benefits from extracellular matrix (ECM). ECM, in the form of tissue‐derived laminin or geltrex, is more effective added to medium during replating rather than pre‐coating.^[^ ^98^, ^103^ ^]^ Geltrex has also been reported to sustain feeder‐free naïve PSCs confined in microfluidics chambers.^[^ ^123^ ^]^ ECM improves attachment of naïve cells and the formation of compact colonies, but mechanistically how it contributes to support naïve PSC identity and self‐renewal is unclear.

The role of autocrine factors NODAL and GDF3 in naïve PSCs should also not be ignored. Downstream signaling through SMAD2/3 is not required in mouse ES cells.^[^ ^124^ ^]^ However, for human naïve PSCs, blockade of the Nodal receptors (ALK5, ALK4, and ALK7) is only tolerated short‐term and eventually leads to trophectoderm differentiation.^[^ ^98^ ^]^ Furthermore, in the absence of XAV939 and Gö6983, inhibition of NODAL signaling combines synergistically with MEK inhibition to promote trophectoderm differentiation and blastoid formation.^[^ ^98^, ^103^ ^]^

As discussed above, production of trophectoderm and hypoblast from epiblast stage cells is antithetical to the developmental sequence of lineage diversification established form mouse blastocyst formation. Transcriptome analyses point to dedifferentiation of naïve PSCs from epiblast to ICM‐like identity prior to differentiation into either extraembryonic lineage.^[^ ^107^, ^125^ ^]^ This raises interesting questions of how reversion is constrained during naïve PSC self‐renewal and whether it may be possible to capture and propagate pre‐epiblast ICM‐like cells.

In summary, while the essential requirements for human naïve PSC self‐renewal are now known and partly rationalized, not all effects are well understood. Further investigation is expected to uncover manipulations of additional pathway(s) that enhance self‐renewal and address limitation of current conditions in which feeder‐free expansion is limited, genomic imprints are erased,^[^ ^126^ ^]^ and XX chromosome status does not fully recapitulate in vivo events.^[^ ^127^ ^]^

## BETWEEN NAÏVE AND PRIMED PLURIPOTENCY

**FIGURE 3:**
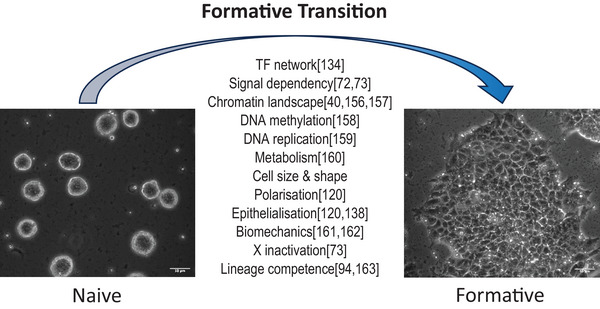
Formative transition – gain of lineage competence. Human naïve pluripotent stem cells (PSCs) withdrawn from PXGL undergo comprehensive reconfiguration of molecular and cellular attributes over several days of formative transition. Images show HNES1 embryo‐derived naïve PSCs in PXGL (left) and after 5 days transition (right). Note the dramatic increase in cell size. Capacitated cells can be expanded long‐term in AFX or other media for primed PSCs and become fully responsive to somatic lineage induction. The formative period relates to the bilaminar disk stage of human embryogenesis (Carnegie stage 5). Scale bar: 50 µM.

Naïve and primed PSC types represent initial and late stages of epiblast that diverge in a multitude of molecular and cell biological features.^[^ ^128^ ^]^ In vivo progression from naïve to primed epiblast proceeds over 2–3 days in the mouse embryo. In the human embryo this process is slower and gastrulation does not commence until 6–7 days after implantation. Non‐responsiveness of human naïve PSCs (counterparts of epiblast at Carnegie Stage 4) to somatic lineage induction is therefore unsurprising without conversion to the equivalent of post‐implantation bilaminar disk epiblast (Carnegie Stage 5). During this 1st week of peri‐ and post‐implantation development the epiblast forms a layer of columnar epithelium overlying the hypoblast and expands in readiness for gastrulation. The term “formative transition” has been coined to describe the multifaceted cellular reorganization, including signaling pathway and enhancer rewiring that are instrumental for gain of lineage competence^[^ ^128^ ^]^ (Figure 3).

On withdrawal of 2iLIF components, mouse ES cell identity becomes extinguished within 24–60 h.^[^ ^129^, ^130^ ^]^ Correspondingly, the ability to derive ES cells from embryo epiblast is lost by the early egg cylinder (E5.5).^[^ ^37^, ^131^ ^]^ Transition from the naïve epiblast state is metachronous in vivo and in vitro.^[^ ^132^, ^133^ ^]^ ES cells undergo a series of changes in cellular and molecular phenotype that are initially reversible if cells are restored to 2iLIF.^[^ ^50^ ^]^ Exit is irreversible at the point when all of the ES cell‐specific pluripotency transcription factors are no longer expressed.^[^ ^130^, ^132^, ^134^, ^135^ ^]^ Post‐implantation epiblast factors such as Otx2, Pou3f1, and Zic3 begin to be upregulated before this point.^[^ ^129^ ^]^ Transcription factor fluctuations seen in serum and LIF may be related to the reversible phase of the transition process. Culture conditions have also been reported that cause entire ES cell populations to adopt more epithelial character and acquire relatively stable shifts in transcription factor expression toward peri‐implantation epiblast.^[^ ^136^, ^137^, ^138^ ^]^ Despite the phenotype shift, these cultures have not fully transitioned, however, because they readily form ES cell colonies when transferred back to serum/LIF or 2iLIF culture.^[^ ^137^, ^138^ ^]^

PSCs beyond the transition point can be derived from early post‐implantation epiblast (E5.5) as well as from ES cells. Such formative‐type stem cells are maintained in activin and XAV939 and require active ERK signaling.^[^ ^139^, ^140^ ^]^ They are unable to form ES cell colonies in 2iLIF but have not progressed to become EpiSCs and are transcriptomically more similar to E5.5 than either earlier or later epiblast.^[^ ^130^, ^139^ ^]^ Unlike mouse EpiSCs, they respond to in vitro germ cell induction, and they can make low level contributions to chimeras.

Intermediate phenotype PSCs have also been reported in human.^[^ ^136^, ^139^, ^141^ ^]^ However, classification of human formative‐type PSCs is more challenging. Only historical histological specimens exist for peri‐ or early post‐implantation epiblast. Information from non‐human primates^[^ ^74^ ^]^ or from extended in vitro development of human embryos^[^ ^142^ ^]^ provide useful but uncertain benchmarks. Furthermore, it is unlikely that the formative transition proceeds in the same manner as in mouse. Not only is the timescale considerably longer, loss of extraembryonic lineage capacity and early gain of competence for amnion (the first post‐implantation lineage in primates) do not occur in mouse epiblast or ES cells. Moreover, competence for germline and of the somatic lineages may not develop simultaneously. Instead, there may be a sequence of formative states. Human formative pluripotency and associated PSC states is an ongoing voyage of exploration. A critical challenge is to determine the optimal conditions for capacitation, which in principle should recapitulate the formative transition process in the embryonic disc.

## PERSPECTIVE

The pluripotent epiblast is a dynamic and time‐limited lineage in the embryo. Ex vivo, however, signaling environments can be engineered that sustain pluripotency indefinitely, producing self‐renewing PSCs. Propagation of PSCs is dependent on constant provision of a specific signaling environment, without which cells resume their developmental journey to differentiation. Travel can be suspended at various waystations along the lineage trajectory without compromising developmental potency, at least in mouse and human.

The potential for self‐renewal derives from the architecture of the pluripotency gene regulatory network. Given the risk of developmental disruption and tumor formation, what could be the benefit to the embryo of a pluripotent compartment enabled for sustained proliferation? Mammalian embryos are regulative, meaning that cell numbers and fates are not predetermined but self‐organize through cellular interactions.^[^ ^143^ ^]^ Thus, there is variability within and between embryos. Regulative flexibility of fate specification relies on plasticity of pluripotent epiblast. Plasticity also includes cell number regulation which ensures the appropriate epiblast population size for gastrulation, allowing compensation for cell loss^[^ ^144^ ^]^ and adjustment to perturbations, notably development of monozygotic twins after embryo splitting, and chimera formation by incorporation of introduced cells.^[^ ^145^ ^]^ These regulative phenomena require that the proliferative lifespan of epiblast cells is not strictly fixed. Nonetheless, epiblast proliferation is constrained in the embryo and ultimately terminated by enforced differentiation. In contrast, latent potential for continuous propagation can be unchained in vitro.

Importantly, however, if the signaling environment is deficient or lacks precision, cells may shift along, or deviate from, the developmental pathway of epiblast. This can result in heterogeneous cultures and/or drift away from epiblast identity.^[^ ^21^, ^83^, ^146^, ^147^ ^]^ Moreover, like any other in vitro cell culture, PSCs are both genetically mutable and susceptible to epigenomic adaptations.^[^ ^148^, ^149^, ^150^ ^]^ Therefore, it always has to be borne in mind that the culture environment is artificial and may select for, or induce, aberrant cell genotypes and phenotypes.

Thus, for any PSC culture there are four basic issues to characterize and understand:

- phenotypic and functional relatedness to pluripotent cells resident in the embryo
- cellular heterogeneity and associated dynamics and/or hierarchy
- integrity and stability of the genome and epigenome
- mechanisms and developmental logic of self‐renewal and differentiation signaling.

The past half‐century has seen remarkable discoveries in the field of PSC biology that have transformed mammalian developmental biology and created a new arena of biomedical and translational opportunity. Some of the exciting challenges remaining are highlighted above. In addition, naïve pluripotency remains a frontier for most species. Currently primed type PSCs have been established for various mammals, but naïve PSCs are limited to rodents and primates. Environments for sustaining naïve PSC self‐renewal differ for mouse and human as discussed above, and formulae are adjusted even between mouse and rat.^[^ ^151^, ^152^ ^]^ Divergence in key transcription factor expression is evidence of evolutionary drift in the naïve pluripotency gene regulatory network that can result in altered signaling requirements.^[^ ^153^, ^154^ ^]^ Moreover, the potential for unlimited self‐renewal at the naïve stage may not exist in all mammals or in other amniotes because it is not a phase of significant cell proliferation in vivo, unlike formative or primed epiblast. On the other hand, it can be argued that naïve pluripotency is a unique state that is the common foundation for germline and soma in all mammals and may therefore be expected to have shared underlying regulatory features. While the facility for diapause may be a predisposing factor for naïve PSC establishment in rodents, it is not an essential pre‐condition. Notably, for both rodent ES cells and human naïve PSCs, proliferation appears contingent only on appropriate nutrients.^[^ ^155^ ^]^ If that is an intrinsic feature of the naïve pluripotency network across species, the challenge of establishing naïve PSCs simplifies to curtailing differentiation. The generation and characterization of naïve PSCs from diverse eutherians, marsupials, monotremes, birds, and reptiles remains a fascinating quest that may uncover a molecular rubric for pluripotency.

## CONFLICT OF INTEREST STATEMENT

The author is an inventor on a patent application filed by the University of Cambridge relating to human naive pluripotent stem cells.

## Data Availability

Data sharing is not applicable to this article as no new data were created or analyzed in this study.
